# aCGH Analysis Reveals Novel Mutations Associated with Congenital Diaphragmatic Hernia Plus (CDH+)

**DOI:** 10.3390/jcm12196111

**Published:** 2023-09-22

**Authors:** Yannick Schreiner, Teresa Stoll, Oliver Nowak, Meike Weis, Svetlana Hetjens, Eric Steck, Alba Perez Ortiz, Neysan Rafat

**Affiliations:** 1 Department of Neonatology, University Children’s Hospital Mannheim, University of Heidelberg, 69117 Mannheim, Germany; yannickalexander.schreiner@umm.de (Y.S.); teresa.stoll@t-online.de (T.S.); alba.perez-ortiz@umm.de (A.P.O.); 2 Department of Gynecology and Obstetrics, University Hospital Mannheim, University of Heidelberg, 68167 Mannheim, Germany; oliver.nowak@umm.de; 3 Department of Clinical Radiology and Nuclear Medicine, University Medical Center Mannheim, University of Heidelberg, 69117 Mannheim, Germany; meike.weis@umm.de; 4 Department of Medical Statistics and Biomathematics, Medical Faculty Mannheim, University of Heidelberg, 69117 Mannheim, Germany; svetlana.hetjens@medma.uni-heidelberg.de; 5 SYNLAB Centre for Human Genetics, 68163 Mannheim, Germany; eric.steck@synlab.com; 6 Department of Neonatology, Center for Children, Adolescent and Women’s Medicine, Olgahospital, Klinikum Stuttgart, 70174 Stuttgart, Germany

**Keywords:** congenital diaphragmatic hernia plus, array-based genomic hybridization, copy number variations, single nucleotide variations

## Abstract

Congenital diaphragmatic hernia (CDH) is a major birth anomaly that often occurs with additional non-hernia-related malformations, and is then referred to as CDH+. While the impact of genetic alterations does not play a major role in isolated CDH, patients with CDH+ display mutations that are usually determined via array-based comparative genomic hybridization (aCGH). We analyzed 43 patients with CDH+ between 2012 and 2021 to identify novel specific mutations via aCGH associated with CDH+ and its outcome. Deletions (*n* = 32) and duplications (*n* = 29) classified as either pathological or variants of unknown significance (VUS) could be detected. We determined a heterozygous deletion of approximately 3.75 Mb located at 8p23.1 involving several genes including *GATA4*, *NEIL2*, *SOX7*, and *MSRA*, which was consequently evaluated as pathological. Another heterozygous deletion within the region of 9p23 (9,972,017-10,034,230 kb) encompassing the *Protein Tyrosine Phosphatase Receptor Type Delta* gene (*PTPRD*) was identified in 2 patients. This work expands the knowledge of genetic alterations associated with CDH+ and proposes two novel candidate genes discovered via aCGH.

## 1. Introduction

Congenital diaphragmatic hernia (CDH) is a life-threatening condition occurring in approximately 1 of 4000 live births [1]. Most cases occur sporadically, referred to as isolated or nonsyndromic, but a proportion of cases of CDH can also be accompanied by additional malformations, e.g., genitourinary, renal, cerebral, or cardiac anomalies, which is then referred to as syndromic CDH or CDH with non-hernia-related anomalies (CDH+). They may account for as much as 50% of all CDH cases, though the exact incidence has been difficult to determine because of unequal definition criteria and the correct identification of additional anomalies [2,3]. Whilst in general, survival of CDH-affected children has greatly improved over the past decades, the prognosis of CDH+-affected children remains poor [2]. Additionally, long-term morbidity mainly resulting from chronic lung disease (CLD) and extracorporeal membrane oxygenation (ECMO)-associated adverse effects remains an important issue. In recent years, tremendous effort has been pursued to investigate the contributions of genetics to the pathogenicity of CDH development. Today, we possess comprehensive lists and summaries of a plethora of genetic variations associated with CDH [4,5,6]. These range from polyploidy to complex chromosomal aberrations such as copy number (CNVs) or single nucleotide variations (SNVs) either being discovered by array-based comparative genomic hybridization (aCGH), whole exome sequencing (WES), or deep gene sequencing. Only a few of them seem likely to be associated with the development of the diaphragm based on our current understanding of molecular networks and signaling pathways throughout organogenesis. For example, molecules acting in retinol-pathways [7,8,9,10] or members of *GATA4/FOG2*-signaling which seem to be crucial for the non-muscular part of the premature diaphragm to evolve can relatively coherently be identified as definitive contributors to CDH [11,12,13,14,15]. Most of the described genetic anomalies, however, lack immediate association to diaphragm development; hence, their potential contribution to its pathogenicity can only be assumed with varying plausibility. Yet, the growing evidence of a genetic influence on CDH development remains important. In the current project, we set out to identify novel specific mutations via aCGH associated with CDH+ and examine whether statements on the clinical outcome of the patient can be made based on the severity of the expected malformation.

## 2. Materials and Methods

### 2.1. Study Cohort and Diagnostic Procedures

We conducted a retrospective study to investigate genetic anomalies associated with CDH. Additionally, we retrospectively compared clinical outcome parameters between CDH+ and CDH− cohorts. All newborns with CDH born between June 2012 and February 2021 who were treated at our neonatal intensive care unit (NICU) at the Department of Neonatology of the University Children’s Hospital Mannheim, University of Heidelberg, were screened for inclusion. Inclusion criteria for the study population were CDH+, namely patients with associated anomalies (including cardiac malformation), syndromes, or chromosomal aberrations. Exclusion criteria were patients with isolated CDH (CDH−) or missing genetic testing/data. The study was conducted in accordance with the Declaration of Helsinki and approved by the local ethics committee of the Medical Faculty Mannheim of the University of Heidelberg (reference number: 2019-885R). This is a retrospective evaluation only of already existing data material. No additional examinations or determinations were made. The data evaluation was carried out in a pseudonymous way.

The study population was then retrospectively compared to a control group consisting of *n* = 44 randomly selected newborns with isolated CDH (CDH−) from our internal CDH registry. Our dataset inquiry included sex, birth weight, 5-min APGAR, side of CDH, and if fetoscopic tracheal occlusion (FETO) was performed. Further clinical parameters collected included prenatal screening parameters such as head circumference, biparietal diameter, abdomen circumference, femur length, and the presence of polyhydramnion. The longest diameter method was used to evaluate LHR and o/e LHR and values were calculated according to a reference formula as described previously [16]. Furthermore, relative fetal lung volumes were assessed. Regarding the CDH characteristics, intraoperative findings were recorded to ensure definitive and reliable records. For those children who had not been undergoing surgical intervention, prenatal magnetic resonance imaging (MRI) findings were taken into consideration to describe the herniated viscera. The pulmonary hypertension of newborns was routinely assessed via echocardiography. For those children who died shortly after delivery, severe pulmonary hypertension was presumed if differences between pre- and postductal oxygen saturation exceeded 10%. Concerning therapeutical interventions, the durations of ECMO and mechanical ventilation (MV) were measured, if applied. For outcome analysis, the incidence of chronic lung disease (CLD) as described previously [17,18] and its severity and overall survival were assessed.

### 2.2. Array-Based Comparative Genomic Hybridization Analysis

In every case of CDH+ at our department, genetic analysis via aCGH is offered to the parents and informed consent is obtained. In addition, copy number variants were also investigated in parental samples if available to determine inheritance and were considered de novo if absent therein. Genetic diagnostics were performed with our cooperation partner (SYNLAB MVZ Humangenetik GmbH, Mannheim, Germany) using CGX™-HD arrays (PerkinElmer, Waltham, MA, USA) with approximately 180,000 oligos covering the entire human genome (mean probe spacing 10 kb in clinically relevant regions and 20 kb in other regions). Cytogenomics software version 2.5.8.11 (Agilent Technologies, Santa Clara, CA, USA) and Genoglyphix version 3.3 (PerkinElmer, Waltham, MA, USA) were used for the evaluation of results. Genomic anomalies were considered significant if at least five adjacent probes with a mean deviation of ≥0.35 or ≤−0.5 (log2 ratio) could be detected compared to the control probe allowing a detectability threshold of approximately 50 kb. HG19 Genome Reference Consortium GRCh37 was used as a reference sequence. Benign Copy Number Variations (CNVs) and CNVs that do not affect protein-coding genes are not reported.

### 2.3. Data Analysis

Categorical variables are presented as percentages whereas continuous variables are presented as means ± standard deviation (SD). To compare groups regarding qualitative parameters, a Chi-square test or Fisher’s exact test was used, where appropriate. The mean values of two groups were compared by two-sample *t*-tests (in the case of normally distributed data) or the Mann–Whitney U-test. A *p*-value < 0.05 was considered statistically significant. Statistical calculations were performed using SAS software version 9.4 (SAS Institute Inc., Cary, NC, USA).

## 3. Results

### 3.1. Patient’s Characteristics

**Figure 1:**
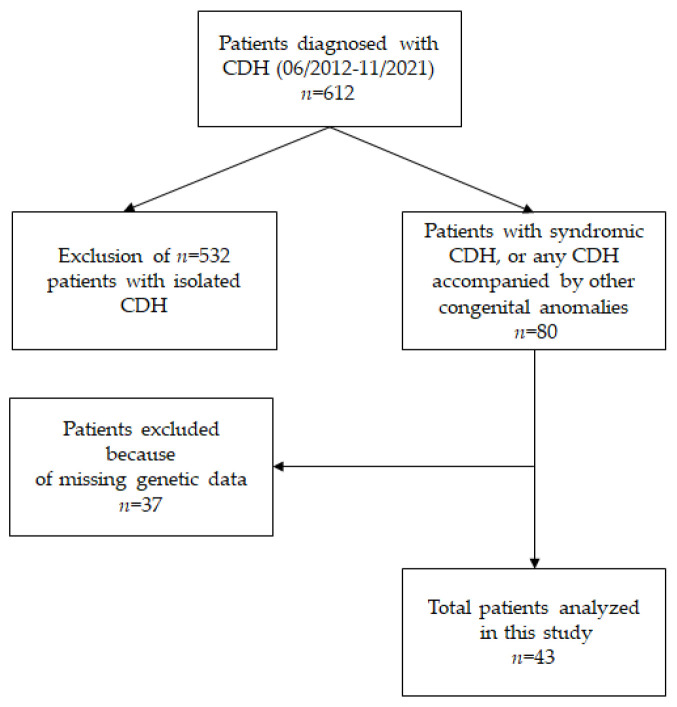
Flow chart representing the composition of the study population.

In the period of 06/2012 to 11/2021, *n* = 80 newborns with CDH+ were treated at our hospital. aCGH results could be obtained from *n* = 43 patients, of whom 16 (37.2%) were male and 27 (62.8%) were female. Figure 1 visualizes the screening process for the study set-up.

**Figure 2:**
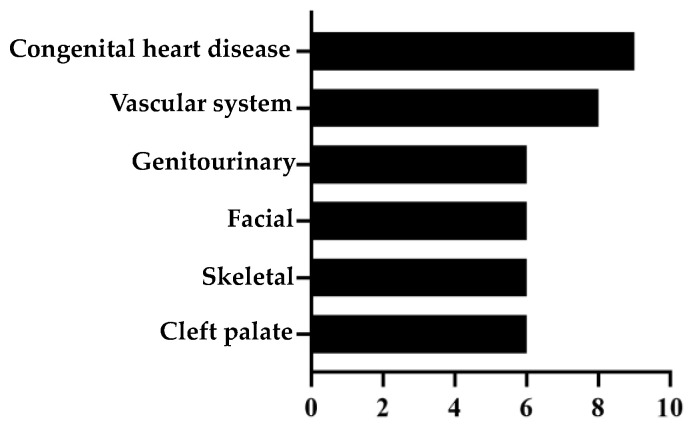
Most frequently observed additional malformations in children diagnosed with syndromic CDH.

Amongst commonly observed additional malformations, congenital heart disease (atrium septum defects (ASD), ventricle septum defects (VSD), tetralogy of Fallot and anomalies of the vascular system (aortic coarctation, hypoplastic aortic arch, truncus arteriosus communis, agenesis of the inferior vena cava, continuity of the hemiazygos vein) were most frequent (Figure 2). Interestingly, one child with CDH+ was also diagnosed with pulmonary capillary hemangiomatosis.

Three female neonates were diagnosed with either Angelman’s Syndrome, Silver–Russel Syndrome, or mosaic trisomy 16, respectively. In 2 cases, the parents were consanguine. In the CDH+ group, right-sided CDH was more frequently observed than in the control group of isolated CDH (*n* = 11 vs. *n* = 2; *p* = 0.002). Notably, in 3 cases (6.98%), bilateral defects could be determined in the CDH+ group whereas no child of the control group presented with such defects. Table 1 summarizes the population data of both the study group (CDH+) and the control group (CDH−).

### 3.2. Prenatal Evaluation of Congenital Diaphragmatic Hernia

Polyhydramnion was determined in *n* = 17 (39.5%) children with CDH+ and in *n* = 16 (36.4%) children with isolated CDH (*p* = 0.41).

As standard parameters evaluated within prenatal ultrasound, LHR, o/e LHR, and rLV were measured or calculated. Significant lower values could be determined for the CDH+ group regarding LHR (1.36 ± 0.40 vs. 1.68 ± 0.51, *p* = 0.007) and rLV (26.9 ± 11.8 vs. 36.2 ± 14.8, *p* = 0.0162), whereas o/e LHR values did not differ significantly (33.4 ± 12.3 vs. 39.3 ± 12.3, *p* = 0.075).

### 3.3. Novel CNVs in Cases of Syndromic CDH

aCGH results were available for 43 CDH+ patients. Results were unremarkable in *n* = 11 cases. In total, *n* = 32 deletions and *n* = 29 duplications classified as either pathological or variants of unknown significance (VUS) could be detected. A detailed compilation of all genetic results and associated anomalies is displayed in Supplementary Table S1. Recurrent mutations are shown in Table 2. First, we determined a heterozygous deletion of approximately 3.75 Mb located at 8p23.1 involving several genes including GATA4, NEIL2, SOX7, and MSRA which was consequently evaluated as pathological. This patient (CDH_033) exhibited a long distance aortic coarctation along with left-sided CDH. Another, much shorter, heterozygous deletion of only 28.5 kb at 8p23.1 could be determined in a second male patient (CDH_004), who was diagnosed with severe pulmonary hypertension following left-sided CDH and presented with cholestasis and liver infarction with unclear etiology. This mutation, however, encompassed neither GATA4 nor any other known candidates but only MSRA. Notably, in the same individuum, there was also a deletion of 9q34.3 encompassing PNPLA7 and MRPL41 whose significance remains uncertain.

**Figure 3:**
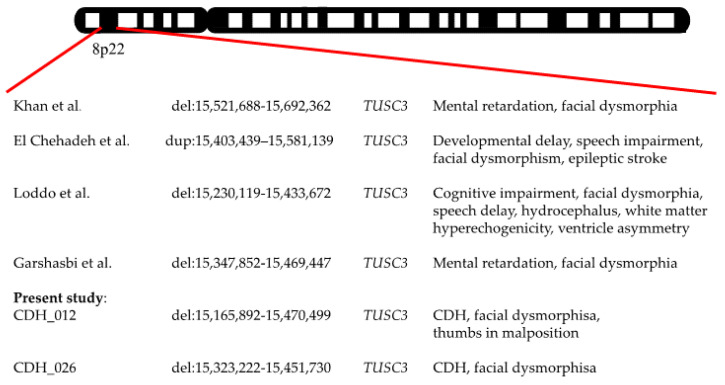
Known mutations within 8p22 associated with CDH. Novel mutations are highlighted [19,20,21,22].

**Figure 4:**
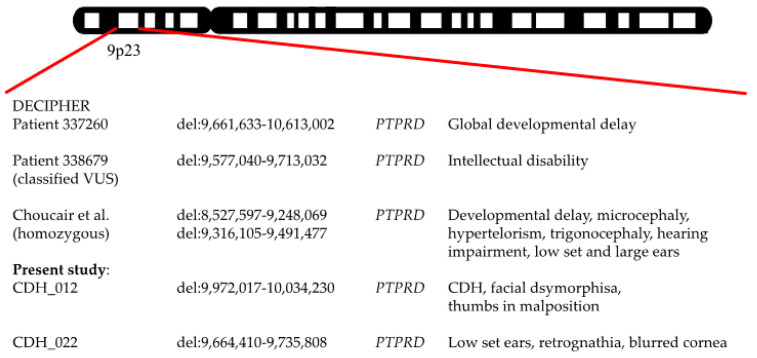
Known mutations within 9p23 associated with CDH. Novel mutations are highlighted [23].

Suspicious results involving a partial deletion of approximately 304,607 kb and 128,508 kb, respectively, located at 8p22 affecting TUSC3 could be obtained from two female patients (CDH_012 and CDH_026) (Figure 3). In each case, the hernia was located on the left side and both neonates exhibited facial anomalies in addition. Both suffered from severe pulmonary hypertension and died after 4 and 6 days of hospitalization, respectively. Furthermore, patient CDH_012 exhibited another heterozygous deletion within the region of 9p23 (9,972,017-10,034,230 kb) (Figure 4) encompassing the Protein Tyrosine Phosphatase Receptor Type Delta gene (PTPRD) classified as a variance of unknown significance. Additionally, this gene was also partially deleted in another female preterm neonate (CDH_022; 9,664,410-9,735,808 kb) with right-sided CDH. She also showed facial dysmorphic features including low set ears, retrognathia, and corneal opacity.

### 3.4. Outcome Parameters of Neonates with CDH+ and CDH−

Duration of MV, duration of ECMO, the incidence of CLD, duration of hospitalization, and overall survival were chosen as outcome parameters to measure morbidity and mortality of CDH+ and isolated CDH cases.

Given the low survival rate of the CDH+ affected neonates (23.3%), outcome parameter measurements were split and calculated separately for both the survivors and the deceased. If looking at the survivors, children with CDH+ exhibited significantly longer duration of MV (33.0 ± 12.5 vs. 25.2 ± 14.7 d, *p* = 0.0357) and slightly longer duration of ECMO (10.4 ± 2.35 vs. 9.85 ± 3.74 d, *p* = 0.8041), based upon which the duration of hospitalization increased accordingly without reaching significance (115 ± 67.6 vs. 60.5 ± 29.0 d, *p* = 0.0698). 

However, if looking at the deceased only, this relation was reversed (Table 1). On the other hand, the survival rate of the control group was fairly high (77.3%). No significant difference regarding the incidence of CLD in survivors could be determined.

## 4. Discussion

In the present study, we performed genetic analyses via aCGH in CDH+ patients to discover novel genetic anomalies and compared clinical outcome parameters between CDH+ and CDH−. We report two novel CNVs affecting *TUSC3* and *PTPRD* that might contribute to CDH development and highlight significant differences in morbidity and mortality between CDH+ and isolated CDH. In our study, we performed aCGH diagnostics in cases of CDH+ to identify novel mutations that might be attributed to CDH development or might even be causally linked to its pathogenicity and considered reoccurring chromosomal aberrations as most promising. For some of those mutations, deletions or duplications contain many different genes and therefore represent major imbalances that might explain all observed malformations for this reason alone. In most cases, however, we could not establish a link between the copy number gains or losses to explain CDH development.

On the other hand, we observed a deletion within 8p23.1 encompassing several genes including *GATA4*, *SOX7*, and *MSRA*. Mutations of this particular region and the genes therein, despite *MSRA*, had been frequently reported in the past and *GATA4* and its regulator encoded by *SOX7* had been suggested to be responsible for CDH development [24,25,26]. We therefore consider the variant of 8p23.1 involving *GATA4* and *SOX7* as causative for the corresponding CDH phenotype. However, although *MSRA* was affected by deletions previously described [24,27], its potential contribution to CDH development has not been discussed before. We demonstrate another patient exhibiting a significant *MSRA* deletion evaluated as VUS along with a deletion of 9q34.3 encompassing *PNPLA7* and *MRPL41* whose significances remain unclear. *MSRA* encodes a ubiquitously expressed and highly conserved enzyme which reduces methionine sulfoxide to methionine, thereby maintaining adequate protein folding [28]. Interestingly, *MSRA*-deficiency has been linked to increased acetaminophen-induced liver injury [29] and might therefore explain cholestasis and liver infarction that we observed in the affected neonate. To date, we are unable to provide a possible connection to CDH development. Anomalies related to deletions involving the genes *GATA4*, *NEIL2*, *SOX7*, and *MSRA* might not be limited to CDH. However, we are unable to explain the long aortic coarctation exhibited by this individual based on its genotype. Furthermore, our analysis revealed novel CNVs in *TUSC3* putatively associated to CDH development that, to our knowledge, had not been described before.

Both duplications and deletions of 8p22 encompassing *TUSC3* had previously been described and linked to familial cases of mental retardation, speech impairment, learning disabilities, and facial dysmorphia [19,20,21,22]. Interestingly, both of our CDH+ patients carrying the heterozygous *TUSC3* deletion also showed facial dysmorphic features such as low set ears. *TUSC3* encodes a subunit of the oligosaccharyltransferase (OST) complex which spans the membrane of the endoplasmic reticulum (ER) and is involved in both the posttranslational modification of the amino acid chain entering the ER lumen and Mg^2+^ homeostasis [20]. Several groups have discussed its significance and argued that *TUSC3* might either be important for the modification of nerve tissue-specific proteins or its CNV-associated dysfunction could be compensated for in tissues other than the nervous system. This could explain the neurodevelopmental deficits that are attributed to *TUSC3* mutations [20,30]. Numerous studies report *TUSC3*-mediated tumor progression in various malignancies [31,32]. Although we are unable to provide an explanation of how it could contribute to CDH development, we cannot exclude a role during embryogenesis and suggest CDH might be another phenotypic feature following *TUSC3* deletions. 

There is evidence of the importance of *PTPRD* during embryogenesis and hence for the pathogenicity of the observed deletion within 9p23 encompassing this particular gene in two of our patients. As we learn from the drosophila homolog of *PTPRD*, this member of the tyrosine phosphatase family is crucial for axon guidance [33]. Furthermore, evidence of its importance during mammalian embryogenesis comes from Uetani et al., who generated *Protein Tyrosine Phosphatase Receptor Type Sigma* (*PTPRS*) and -*Delta* (*PTPRD*) double-knockout mice and demonstrated both impaired axon targeting to the developing diaphragm and thinner diaphragms in altered muscle ultra-structure in the sense of a developmental arrest during myotube formation [34]. We hypothesize that this disturbance in muscular/neuronal inter-signaling could make the embryo prone to CDH development. To our knowledge, our study is the first to describe *PTPRD* variants in the context of congenital diaphragmatic hernia. In the DECIPHER database, two patients with heterozygous *PTPRD* mutations are mentioned and phenotypically described with intellectual disability and global developmental delay. Another group reported on a male infant with a homozygous deletion of *PTPRD* exhibiting various facial abnormalities along with hearing impairment and also developmental delay [23]. Therefore, the variants of *PTPRD* could be determined as potential novel CNVs involved in CDH development.

Screening programs are provided for pregnant women whose unborn children are suspected to show any congenital anomaly whatsoever. Due to the often-massive intrathoracic hernia content, congenital diaphragm defects are usually determined reliably within prenatal care. However, identifying CDH+ via prenatal ultrasound can be challenging unless additional anomalies are uncovered. LHR, o/e LHR, rLV, and the presence of polyhydramnions are common parameters assessed via prenatal ultrasound. In our study, we observed significantly lower LHR and rLV values in the CDH+ group compared to the control group, indicating a more severe hypoplasia in CDH+ patients. Therefore, low values within prenatal ultrasound examinations of fetuses with CDH should be handled with suspicion as they might justify taking a closer look at the child to not miss any additional malformation.

Our evaluation of clinical outcome parameters reflecting morbidity and mortality highlights the severe medical condition of children affected by CDH+ + and is in line with previous results pointing towards children’s worse outcome if additional malformations are present [2].

Limitations of our study include an incomplete dataset and a retrospective design. Moreover, low incidences of CDH and especially of CDH+ resulting in small study cohorts recruited over many years limit the conclusions drawn from our study. Additionally, although we are aware of positive aCGH results in cases of isolated CDH, we hypothesized the inclusion of only CDH+ patients in genetic analysis would yield the most striking results. As such, some genetic variations might have escaped our notice. Indeed, whole exome sequencing (WES) has become a promising tool to investigate genetic anomalies on the single nucleotide level and it could probably identify novel candidates if primarily aCGH results remain negative. Following the recommendations of Scott et al., we also understand WES as a complementary test and as an escalation of a stepwise diagnosis of syndromic CDH, which now needs to be applied in those individuals showing ‘unremarkable’ results in our aCGH analysis [35].

## 5. Conclusions

Our clinical data confirm the longer need for both MV and ECMO and consequently increased duration of hospitalization in survivors of CDH+. Additional evidence is provided by the relation of outcome parameters between CDH+ and CDH− being reversed upon investigation of deceased children only. We hypothesize that this observation could be explained by the fatal medical conditions and poorer prognosis of children affected by CDH+ and early discontinuation of invasive therapy. Furthermore, we expand our knowledge on molecular networks in the pathogenesis of CDH by contributing novel CNVs associated with CDH+ to our reference list: we propose *PTPRD* and *TUSC3* as novel candidate genes to contribute to CDH development.

## Figures and Tables

**Table 1:** Characterization of the study population.

	CDH+		CDH−		*p*-Value
**All, *n (%)***	43	(100)	44	(100)	
Sex male	16	(37.2)	23	(52.3)	-
CDH left	29	(67.4)	42	(95.5)	0.002
CDH bilateral	3	(6.98)	0	(0.00)	-
**Birth parameters, *Mean ± SD***					
Gestational age [weeks]	36.1		37.8		<0.001
Gestational weight [g]	2387	±707	3114	±443	<0.0001
5 minute APGAR, *Median*	6		8		<0.0001
**Prenatal parameters**					
o/e LHR [%]	33.4	±12.3	39.3	±12.3	0.08
LHR	1.36	±0.40	1.68	±0.51	<0.01
rLV [%]	26.9	±11.8	36.2	±14.8	<0.05
Polyhydramnion, *n (%)*	17	(39.5)	16	(36.4)	0.41
FETO, *n (%)*	4	(9.30)	0	(0.00)	0.05
**CDH characteristics, *n (%)***					
Upside stomach	26	(60.5)	30	(68.2)	0.69
Upside gut	34	(79.1)	43	(97.7)	0.59
Upside liver	34	(79.1)	30	(68.2)	<0.05
Upside kidney	0	(0.00)	0	(0.00)	-
Upside spleen	10	(23.3)	33	(75.0)	<0.01
**Clinical parameters, *n (%)***					
Surgery performed	15	(34.9)	37	(84.1)	<0.0001
ECMO required	18	(41.9)	25	(56.8)	0.16
iOI, *Mean ± SD*	24.6	±9.33	13.9	±11.6	<0.05
**Outcome parameters, *Mean ± SD***					
Duration of ventilation, survivors only [d]	33.0	±12.5	25.2	±14.7	<0.05
Duration of ventilation, deceaesd only [d]	8.40	±12.7	29.3	±48.3	<0.01
Duration of ECMO, survivors only [d]	10.4	±2.35	9.85	±3.74	0.80
Duration of ECMO, deceased only [d]	10.2	±7.16	11.0	±3.61	0.77
Hospitalization, survivors only [d]	115	±67.6	60.5	±29.0	0.07
Hospitalization deceased only [d]	14.2	±36.5	29.1	±48.2	<0.05
CLD in survivors, *n (% of survivors)*	6	(14.0)	18	(40.9)	0.21
Overall survival, *n (%)*	10	(23.3)	34	(77.3)	<0.0001

CDH = congenital diaphragmatic hernia, o/e LHR = observed/expected lung-to-head-ratio, rLV = relative fetal lung volume, FETO = fetal tracheal occlusion, ECMO = extracorporeal membrane oxygenation, CLD = chronic lung disease, iOI = initial oxygenation index.

**Table 2:** Novel genetic findings in CDH+ cases.

Patient-ID	Sex	aCGH Result		Suspected Gene	Inheritance	Evaluation	Additional Anomalies
CDH_012	female	del(8p22)	15,165,892-15,470,499	*TUSC3*	n/a	patho.	Facial abnormalities, thumbs in malposition
CDH_026	female	del(8p22)	15,323,222-15,451,730	*TUSC3*	n/a	patho.	Low set ears, dysmorphia
CDH_004	male	del(8p23.1)	10,091,679-10,120,229	*MSRA*	not paternal	VUS	SGA, cholestasis, liver infarction
CDH_033	female	del(8p23.1)	8,108,992-11,858,460	*GATA4*, *SOX7*	n/a	patho.	Long distance aortic coarctation
CDH_012	female	del(9p23)	9,972,017-10,034,230	*PTPRD*	n/a	VUS	Facial abnormalities, thumbs in malposition
CDH_022 *	female	del(9p23)	9,664,410-9,735,808	*PTPRD*	n/a	VUS	Low set ears, retrognathia, blurred cornea

* right-sided CDH. CDH = congenital diaphragmatic hernia, n/a = not applicable, SGA = small for gestational age, VUS = variation of unknown significance.

## Data Availability

Data are contained within the article or supplementary material.

## Supplementary Materials

The following supporting information can be downloaded at: https://www.mdpi.com/article/10.3390/jcm12196111/s1, Table S1: CNVs determined in patients with CDH+.
