# Supplementary material for: aCGH Analysis Reveals Novel Mutations Associated with Congenital Diaphragmatic Hernia Plus (CDH+)
Source: J Clin Med. 2023 Sep 22;12(19):6111. doi: 10.3390/jcm12196111 (PMC10573849; doi:10.3390/jcm12196111)
Supplement: Supplementary file 1 [file jcm-12-06111-s001.zip › jcm-2620792-supplementary.pdf]

Supplementary Table S1: CNVs determined in patients with CDH+.

	Sex	aCGH Result	Mutation Locus	Candidate Genes	Inheritance	Evaluation	CDH-side	Additional anomalies / clinical observations
CDH_001	m	unremarkable	/	/	/	/	right	Cleft palate
CDH_002	m	dup(16p13.11)	chr16:15,053,284-16,287,900	19 genes affected	not paternal	patho.	left	Hypoplastic aortic arch, dysmorphia
CDH_003	m	unremarkable	/	/	/	/	left	Kryptorchism, bilateral inguinal hernia, large liver
CDH_004	m	del(8p23.1)	chr8:10,091,679-10,120,229	MSRA	not paternal	VUS	left	SGA, liver infarction, cholestatsis
		del(9q34.3)	chr9:140,408,143-140,447,808	PNPLA7, MRPL41	not paternal	VUS		
CDH_005	f	unremarkable	/	/	/	/	right	Radial aplasia
CDH_006	f	dup(11p13)	chr11:33,111,101-33,498,412	CSTF3, CSTF3-AS1, HIPK3	de novo	VUS	left	Truncus arteriosus communis
CDH_007	f	del(6q16.1)	chr6:95,175,791-95,228,921	no genes affected	n/a	VUS	right	Congenital hydrothorax
		dup(Xq27.2)	chrX:140,353,091-140,756,586	SPANXA2-OT1, SPANXA2, SPANXA1	n/a	VUS		
CDH_008	m	dup(4q13.2)	chr4:66,667,229-66,988,055	no genes affected	paternal	VUS	left	Hydrocephalus, mental retardation
		del(5q33.1)	chr5:152,107,716-152,361,320	no genes affected	paternal	VUS		
		del(6q16.1)	chr6:97,466,938-97,496,515	KLHL32	maternal	VUS		
		dup(12q24.31)	chr12:124,113,899-124,188,960	EIF2B1, GTF2H3, TCTN2	maternal	VUS		
		dup(14q22.3)	chr14:55,634,478-55,683,232	DLGAP5	paternal	VUS		
CDH_009	m	dup(13q22.3)	chr13:77,881,896-78,040,144	MYCBP2	paternal	VUS	right	Multiple skeletal anomalies
CDH_010	m	unremarkable	/	/	/	/	left	Pulmonary capillary hemangiomatosis
CDH_011	f	unremarkable	/	/	/	/	left	Cleft palate
CDH_012	f	del(8p22)	chr8:15,165,892-15,470,499	TUSC3	n/a	patho.	left	Facial dysmorphia, enlarged thumbs in malposition
		del(9p23)	chr9:9,972,017-10,034,230	PTPRD	n/a	VUS		
CDH_013	f	del(2q13)	chr2:112,690,985-112,784,018	MERTK	n/a	patho.	left	Syndaktyly, IUGR
		del(5p15.2)	chr5:13,793,439-14,172,157	DNAH5, TRIO	n/a	patho.		

CDH_014	f	dup(1q44)	chr1:245,123,824-245,665,521	LOC101928068, EFCAB2, KIF26B	n/a	VUS	left	Microcephaly, cleft palate
		dup(5q14.3)	chr5:83,095,989-83,185,423	no genes affected	n/a	VUS		
		dup(Xp22.2)	chrX:15,675,429-15,717,134	TMEM27, CA5BP1	n/a	VUS		
CDH_015	m	dup(1p33)	chr1:46,813,401-46,920,381	NSUN4, FAAH, FAAHP1, LINC01398	maternal	VUS	left	Aortic coarctation, renal insufficiency
		del(2q13)	chr2:110,862,369-110,977,999	MALL, NPHP1, LINC00116	paternal	patho.		
		del(4q31.1)	chr4:140,373,276-140,460,459	RAB33B, SETD7	paternal	patho.		
CDH_016	f	dup(3p26.3)	chr3:2,214,694-2,781,212	CNTN4	paternal	VUS	left	Silver-Russel-Syndrome, c.868C>T; p.Arg290Cys in HNF4A
CDH_017$^{\Delta}$	f	dup(1q42.3)	chr1:235,414,132-235,746,839	ARID4B, GGPS1, TBCE, B3GALNT2, GNG4	n/a	VUS	left	Second sibling with CDH in family history
CDH_018$^{\Delta}$	f	del(7p15.3)	chr7:24,335,739-24,411,512	NPY	not maternal	VUS	left	Prenatally suspected mosaic trisomy 16 (verified postnatally!), Growth retardation
		dup(14q23.2)	chr14:64,054,041-64,300,399	WDR89, SGPP1	maternal	VUS		
		del(Xp22.33)	chrX:1,040,107-1,244,544	no genes affected	maternal	VUS		
		dup(Xp22.33)	chrX:1,557,919-1,611,388	ASMTL, P2RY8	maternal	VUS		
CDH_019	f	dup(17q11.2)	chr17:29,116,494-29,135,178	SUZ12P1	paternal	VUS	left	Omphalocele, renal agenesis, congenital heart disease
CDH_020	f	unremarkable	/	/	/	/	left	Congenital hydrothorax, facial anomalies, low set ears, dystrophia
CDH_021	m	dup(11p14.3)	chr11:25,069,132-25,120,576	LUZP2	n/a	VUS	left	Facial anomalies
		del(14q22.1)	chr14:52,030,636-52,539,077	FRMD6, GNG2, RTRAF, NID2	n/a	VUS		
CDH_022	f	del(9p23)	chr9:9,664,410-9,735,808	PTPRD	n/a	VUS	right	Retrognathia, blurred cornea, low set ears
CDH_023	m	unremarkable	/	/	/	/	left	ASD
CDH_024	f	del(2p16.2-p16.1)	chr2:54,948,819-55,008,613	EML6	n/a	VUS	right	Hypertelorism, crossing-over of fingers
CDH_025	f	dup(1q21.1-q21.2)	chr1:145,388,977-147,726,542	41 genes affected	n/a	patho.	left	Multiple facial and skeletal anomalies
		dup(1q23.2-q23.3)	chr1:160,241,394-160,740,194	11 genes affected	n/a	VUS		

CDH_026	f	del(2p16.3)	chr2:50,865,009-50,928,081	NRXN1	n/a	VUS	left	Low set ears, dysmorphia
		del(8p22)	chr8:15,323,222-15,451,730	TUSC3	n/a	patho.		
		del(11q22.1)	chr11:96,761,189-96,960,782	no genes affected	n/a	VUS		
CDH_027	f	del(15q11.2-q13.1)*	chr15:23,717,628-28,513,166	20 genes (incl. UBE3A, NDN, SNRPN)	n/a	patho.*	right	Meckel´s diverticulum, Angelman´s Syndrom (*corresponding mutation)
		dup(Xp22.33)	chrX:2,059,239-2,173,572	DHRSX	n/a	VUS		
		dup(Xp21.1)	chrX:37,222,167-37,524,396	LANCL3, PRRG1	n/a	VUS		
CDH_028	f	del(Xp22.33)	chrX:2,218,949-2,237,127	DHRSX	n/a	VUS	left+right	Cleft palate, DORV, dysplasia of the corpus callosum
CDH_029	f	del(2q37.1-q37.3)	chr2:234,660,517-242,654,701	78 genes affected (incl. HADC4)	n/a	patho.	left	Cleft palate
		dup(19q13.33-q13.43)	chr19:53,962,000-63,782,949	app. 450 genes affected	n/a	patho.		
		del(Xq28)	chrX:146,688,516-148,834,390	MAGEA8	n/a	VUS		
CDH_030	m	del(15q21.3)	chr15:58,672,866-58,720,301	LIPC	n/a	VUS	left	Kataract and deformity of the iris
CDH_031	m	dup(3q12.2)	chr3:100,347,566-100,445,466	GPR128, TFG	n/a	VUS	left	Tetratolgy of Fallot, cleft palate, renal agenesis
		dup(4q22.3)	chr4:96,128,268-96,147,709	UNC5C	n/a	VUS		
		del(17q24.2)	chr17:64,420,296-64,477,351	PRKCA	n/a	VUS		
CDH_032	f	dup(1q21.1-q21.2)	chr1:146,531,538-147,726,541	app. 18 genes affected	paternal	patho.	left	Tretralogy of Fallot, AVSD, alcohol embryopathy
CDH_033	f	dup(5q13.3)	chr5:76,086,349-76,134,632	F2RL1	n/a	VUS	left	Long distance aortic coarctation
		del(8p23.1)	chr8:8,108,992-11,858,460	42 genes (incl. GATA4, SOX7, MSRA)	n/a	patho.		
CDH_034	m	unremarkable	/	/	/	/	left	Hypoplasia of the aortic arch, small left ventricle
CDH_035	f	del(1p36.23)	chr1:8,703,350-8,712,518	RERE	n/a	VUS	left	Hypoplasia of the aortic arch
CDH_036	f	unremarkable	/	/	/	/	left	VSD
CDH_037	f	del(7q11.22)	chr7:69,309,002-69,323,591	AUTS2	paternal	VUS	left	Cleft palate, anal atresia, renal agenesis, oesophageal atresia
CDH_038	f	unremarkable	/	/	/	/	left	Discontinuity of the aortic arch, VSD, ASD

CDH_039	m	dup(3q28)	n/a	n/a	maternal	VUS	n/a	Open ductus arteriosus, cryptorchism, malrotation of cecum and colon, ASD
		del(8p23.1)	n/a	n/a	not maternal	patho.		
CDH_040	f	del(1p21.2-p13.2)	chr1:101,203,862-114,107,964	app. 134 genes affected	de novo	patho.	left	Aortic coarctation, hypoplastic aortic arch, large ASD II
		del(12q24.31)	chr12:121,259,053-121,280,054	SPPL3	maternal	VUS		
CDH_041	m	unremarkable	/	/	/	/	right	Agenesis of the inferior vena cava, rip fusion, VACTERL
CDH_042	m	del(6q25.1)	chr6:151,859,683-151,896,154	CCDC1170	n/a	VUS	left+right	DORV, ASD
CDH_043	f	dup(16q21-q24.3)	chr16:61,828,999-90,119,719	app. 360 genes affected	n/a	patho.	right	IUGR, left hand in malposition, congenital bilateral hydrothorax
		dup(18p11.32-q12.2)	chr18:141,354-35,399,420	app. 215 genes affected	n/a	patho.		

Note, that all patients were heterozygous for deletions. CDH = congenital diaphragmatic hernia, n/a = not available, VUS = variance of unknown significance, patho. = pathological, SGA = small for gestational age, IUGR = intrauterine growth restriction. VSD = ventricular septum defect, ASD = atrial septum defect, AVSC = combined atrial and ventricular septum defect, DORV = double outlet right ventricle, Δ = consanguine parents.
